# Stimulation of motilin secretion by bile, free fatty acids, and acidification in human duodenal organoids

**DOI:** 10.1016/j.molmet.2021.101356

**Published:** 2021-10-15

**Authors:** Emily L. Miedzybrodzka, Rachel E. Foreman, Van B. Lu, Amy L. George, Christopher A. Smith, Pierre Larraufie, Richard G. Kay, Deborah A. Goldspink, Frank Reimann, Fiona M. Gribble

**Affiliations:** Wellcome Trust – MRC Institute of Metabolic Science, Metabolic Research Laboratories, Addenbrooke's Hospital, Hills Road, Cambridge, CB2 0QQ, UK

**Keywords:** Motilin, Secretion, Human intestinal organoids, Enteroendocrine hormones

## Abstract

**Objective:**

Motilin is a proximal small intestinal hormone with roles in gastrointestinal motility, gallbladder emptying, and hunger initiation. *In vivo* motilin release is stimulated by fats, bile, and duodenal acidification but the underlying molecular mechanisms of motilin secretion remain poorly understood. This study aimed to establish the key signaling pathways involved in the regulation of secretion from human motilin-expressing M-cells.

**Methods:**

Human duodenal organoids were CRISPR-Cas9 modified to express the fluorescent protein Venus or the Ca^2+^ sensor GCaMP7s under control of the endogenous motilin promoter. This enabled the identification and purification of M-cells for bulk RNA sequencing, peptidomics, calcium imaging, and electrophysiology. Motilin secretion from 2D organoid-derived cultures was measured by liquid chromatography-tandem mass spectrometry (LC-MS/MS), in parallel with other gut hormones.

**Results:**

Human duodenal M-cells synthesize active forms of motilin and acyl-ghrelin in organoid culture, and also co-express cholecystokinin (CCK). Activation of the bile acid receptor GPBAR1 stimulated a 3.4-fold increase in motilin secretion and increased action potential firing. Agonists of the long-chain fatty acid receptor FFA1 and monoacylglycerol receptor GPR119 stimulated secretion by 2.4-fold and 1.5-fold, respectively. Acidification (pH 5.0) was a potent stimulus of M-cell calcium elevation and electrical activity, an effect attributable to acid-sensing ion channels, and a modest inducer of motilin release.

**Conclusions:**

This study presents the first in-depth transcriptomic and functional characterization of human duodenal motilin-expressing cells. We identify several receptors important for the postprandial and interdigestive regulation of motilin release.

## Highlights

• Mechanisms of motilin secretion were assessed in a new human duodenal organoid model.
• RNA sequencing identified ion channels and receptors involved in M-cell activity.
• M-cells fired action potentials similar to those in other enteroendocrine cell types.
• Motilin release was triggered by low pH, bile, and free fatty acid receptor-1 ligands.
• Low pH depolarized M-cells and increased calcium through acid-sensing ion channels (ASIC).

## Graphical abstract

**Figure:**
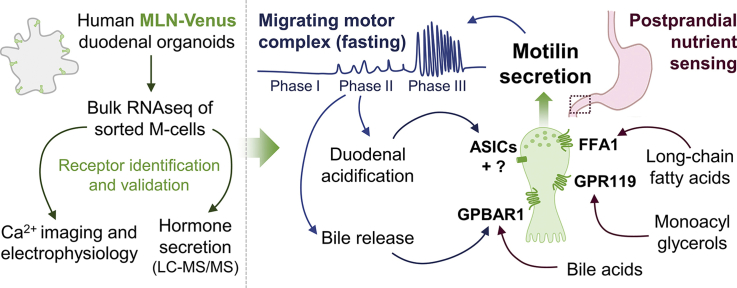

## 1 Introduction

Gut hormones secreted by enteroendocrine cells (EECs) in response to luminal contents and neurohormonal signals coordinate digestion, absorption, nutrient availability, and satiety [1]. Motilin (MLN), a hormone released by the proximal small intestine, has well-established roles in the regulation of gastrointestinal motility and gallbladder emptying [2,3]. Several motilin receptor agonists are under consideration as prokinetic agents for the treatment of gastroparesis, gastro-oesophageal reflux disease, and food intolerance in critically ill patients [[4], [5], [6]]. Motilin has also more recently been implicated in the initiation of hunger, leading to renewed interest in motilin signaling and the underlying mechanisms governing endogenous motilin secretion [7,8].

Circulating motilin levels fluctuate in synchrony with the migrating motor complex (MMC), a pattern of smooth muscle activity which moves from the stomach to the distal small intestine every 1.5 – 2 h during the interdigestive state [9]. The MMC is thought to play a housekeeping role by clearing undigested food, debris, and bacteria from the small intestine [10]. Plasma motilin peaks immediately before the period of strongest peristaltic contractions, known as phase III of the MMC, and exogenous motilin can prematurely induce gastric phase III motor activity [11,12]. It remains unclear how the natural oscillations in motilin levels during the MMC are regulated, although bile and lowered duodenal pH have been proposed to underlie this effect [8,13,14]. Consumption of food results in interruption of the current MMC cycle and postprandial levels of motilin are highly dependent on meal macronutrient composition [15]. Several studies have shown that lipids, given orally or intravenously, stimulate motilin release [[16], [17], [18]].

Although motilin is expressed in most mammals, including humans, the absence of motilin and its receptor, Motilin Receptor (MLNR/GPR38) in laboratory rodent models has hampered the characterisation of motilin physiology compared with other gut hormones [19]. The mechanisms of motilin secretion in the fasted and postprandial states have not previously been studied at a molecular level, owing to a lack of suitable cellular models. An enhanced understanding of endogenous motilin secretion may enable this axis to be targeted in the treatment of gut motility disorders.

In this study, we aimed to label, identify, and purify motilin-expressing M-cells using recently optimized protocols for the labeling of enteroendocrine cells in self-renewing human small intestinal organoid cultures [20]. Through transcriptomic profiling, we revealed candidate receptors and ion channels involved in the regulation of motilin secretion. To enable simultaneous measurement of motilin with other proximal small intestine-derived enteroendocrine hormones, we developed a novel LC-MS/MS multiplexed method to measure the endogenous human motilin peptide, rather than relying on the traditionally employed radio-immunoassay. Using LC-MS/MS alongside single-cell electrophysiology and calcium imaging assays enabled us to characterize several molecular mechanisms underlying fat-, bile- and acid-induced motilin release.

## 2 Materials and methods

### 2.1 Human organoid culture

Human duodenal organoids were generated from fully anonymized specimens provided by Addenbrooke's Hospital Tissue Bank under East of England–Cambridge Central Research Ethics Committee approval (ref: 09/H0308/24). Organoids were generated, maintained, and differentiated as previously described, with passaging every 10–14 days [20]. IGF-1/FGF-2 (IF) human organoid media was used [21], with the addition of Notch (10 μM DAPT, Generon) and MEK (100 nM PD0325901, Sigma–Aldrich) inhibitors in 5% Wnt IF media (termed IF∗) to promote increased M-cell differentiation where required [20].

**Figure 1:**
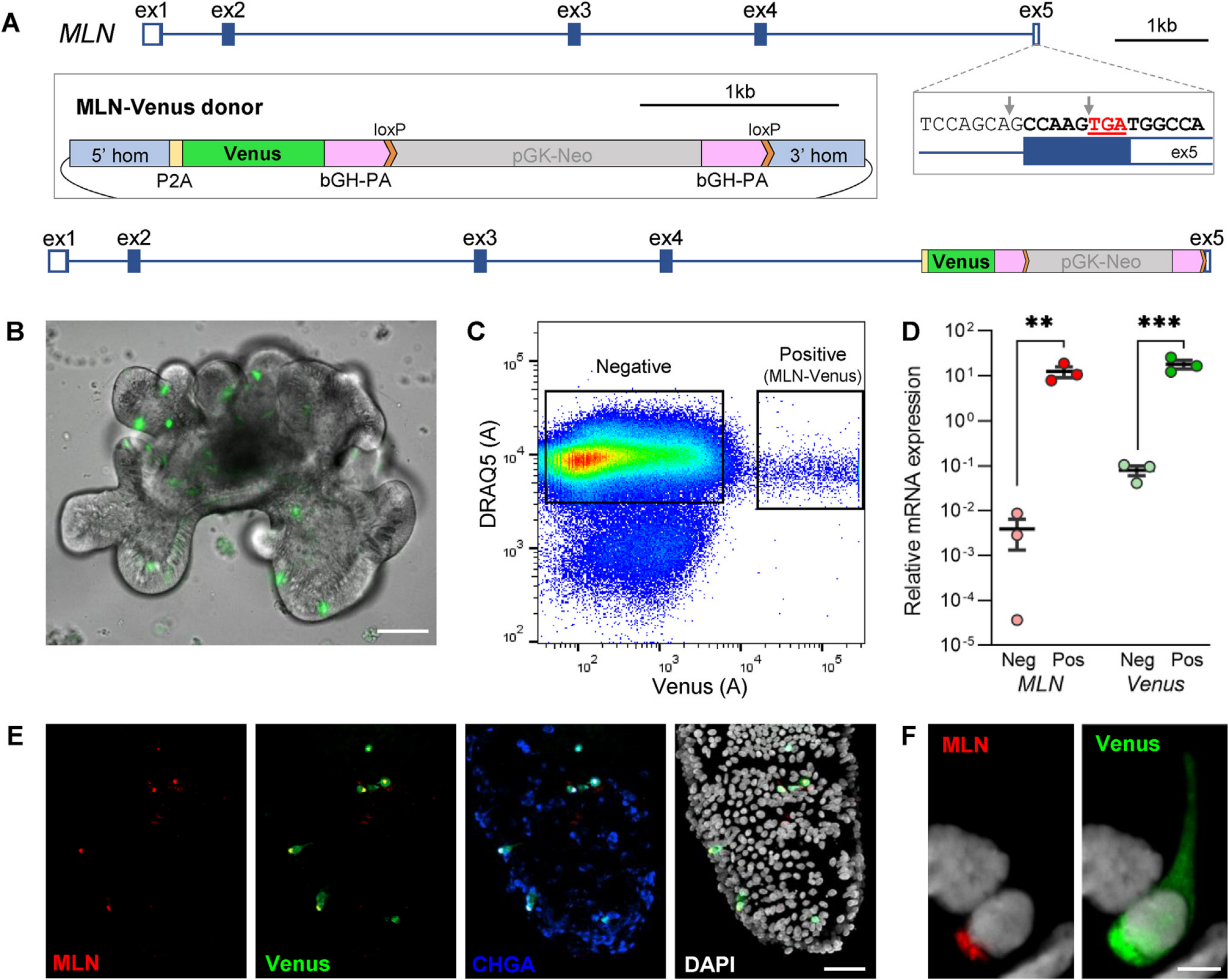
Generation of MLN-Venus human duodenal organoids. **(A)** Schematic showing Venus insertion into the MLN gene by CRISPR-Cas9 homology-directed repair, **(B)** Live image of MLN-Venus human duodenal organoids. Scale bar 100 μm, **(C)** Representative fluorescence-activated cell sort (FACS) plot of 500 k events. MLN-Venus positive and negative populations were collected based on Venus fluorescence, after selecting DAPI-negative, DRAQ5-positive single cells, **(D)** Expression of *MLN* and *Venus* (*YFP*) mRNA in Venus-negative (Neg) and Venus-positive (Pos) sorted cells by qPCR, expressed relative to β-actin. ∗∗p < 0.01, ∗∗∗p < 0.001 by unpaired t-test with Holm-Sidak correction, mean ± SEM presented (n = 3), **(E)** Immunohistochemistry of fixed MLN-Venus human duodenal organoids stained for MLN (red), Venus (GFP, green), and CHGA (blue) with DAPI nuclear stain (white). Maximal z-projection from a confocal stack shown. Scale bar 50 μm, **(F)** Basolateral localization of MLN staining in a single Venus-positive cell. Scale bar 5 μm.

*MLN*-Venus and *MLN*-GCaMP7s reporter organoid lines were generated by CRISPR-Cas9-mediated homology-directed repair. Guides targetting exon 5 of the motilin (*MLN*) locus immediately prior to the stop codon (TTTCTCCAGCAGCCAAGTGA and CGTGGCCATCACTTGGCTGC; Figure 1A) were cloned into plasmids co-expressing nuclear-targeted Cas9 (pX330; Addgene plasmid #42230), using the manufacturer's protocols [22]. Donor plasmids were generated by Gibson cloning (New England Biolabs). Plasmids were concentrated (>2 μg/μl) by ethanol precipitation and delivered to dissociated organoids (30 μg of donor and 20 μg of guide/Cas9 plasmid) by electroporation [23]. Antibiotic selection with 0.5 mg/ml G418 began 4-6 days post-electroporation and surviving organoids were manually picked to establish clonal organoid lines. DNA was extracted using QuickExtract DNA Extraction Solution (Lucigen Corporation, USA), with integration tested by PCR screening and confirmed by Sanger sequencing (Source BioScience).

### 2.2 Immunohistochemistry

Organoids were recovered from the matrix and immunostained as described in [24]. Briefly, organoids were fixed in 4% paraformaldehyde on ice for 45 min and antigen-retrieval performed using sodium citrate pH 6.0 for 2 × 20 min at 80 °C. Primary [chicken anti-GFP/Venus (1/1000, ab13970), mouse anti-MLN (1/100, sc-376,605) and rabbit anti CHGA (1/200, sc-13090)] and secondary [1/500 Alexa Fluor donkey anti-chicken 488 (703-545-155), anti-mouse 555 (A31570) or anti-rabbit 647 (A31573)] antibodies were each incubated in 10% donkey serum with 0.1% Triton X-100 overnight at 4 °C. Nuclei were stained with 4′,6-diamidino-2-phenylindole (DAPI, 2 μg/ml) and organoids cleared using 2.5 M fructose in 60% glycerol before mounting on slides and imaging with a Leica SP8 confocal microscope.

### 2.3 Fluorescence-activated cell sort (FACS)

Cell sorting was performed as described previously [25]. Briefly, differentiated organoids grown in IF (bulk RNA sequencing) or IF∗ media (peptidomics) were enzymatically and mechanically digested to single cells. Cells were resuspended in Hanks’ Balanced Salt Solution (without Ca^2+^ or Mg^2+^) supplemented with 10 μM Rho-kinase (ROCK) inhibitor Y-27632 and 10% fetal bovine serum (RNA extraction) or 0.1% bovine serum albumin (peptide extraction). Live DAPI-negative, DRAQ5-positive single cells were sorted based on Venus fluorescence using a FACS Melody cell sorter (BD Biosciences). Venus positive and negative cells (1–18 × 10^3^ cells per sample) were collected in 350 μl RLT + buffer (Qiagen) supplemented with 1% β-mercaptoethanol for RNA extraction, or 250 μl 6 M guanidine hydrochloride for peptidomics.

### 2.4 Quantitative PCR (qPCR) and bulk RNA sequencing

RNA was extracted using RNAeasy Micro Plus kit (Qiagen) and quantified using RNA 6000 Pico Kit and Bioanalyser 2000 (Agilent). qPCR was performed on complementary DNA (cDNA) prepared with SuperScript IV Reserve Transcriptase (Invitrogen), using the following TaqMan probes: *ACTB*, Hs01060665_g1; *MLN*, Hs00757713_m1; *YFP/Venus*, Ac04097229_mr. cDNA libraries were generated from 4 ng input RNA (RIN 7–9) using the SMARTer Stranded Total RNA-Seq v2 Pico Input Mammalian kit (Takara Bio) with thirteen PCR amplification cycles. Libraries were pooled and single-end 50 bases sequenced on a HiSeq 4000 (Illumina).

Quality and adaptor trimming of sequenced transcripts was performed using cutadapt (v2.7). STAR (v2.7.3a) was used to align transcripts to the human genome (GRCh38). Raw counts were generated using featureCounts (v2.0.0). Quality control was performed using FastQC (v0.11.9). Differential gene expression analysis was performed in RStudio using DESeq2 (v1.24.0). Gene annotation was obtained from the Ensembl dataset held in BioMart (v2.40.5). Receptor and ion channel lists were generated from the International Union of Basic and Clinical Pharmacology (IUPHAR) “targets and families” list (Accessed on 7 Jan 2020). RNA sequencing data were deposited in the National Center for Biotechnology Information-Gene Expression Omnibus (NCBI GEO) repository (GSE176552).

### 2.5 Peptidomic analysis of the sorted cells

Sorted cells in 6 M guanidine hydrochloride were subjected to three freeze-thaw events. Lysates were then dried for 16 h in a rotary evaporator under aqueous conditions at room temperature. Samples were reconstituted in 500 μL 0.1% formic acid in water (v/v) for peptide extraction using an HLB PRiME μElution solid-phase extraction plate (Waters) and analyzed following reduction and alkylation using an Ultimate 3000 nano-LC system (Thermo Scientific) coupled to a Q Exactive Plus Orbitrap mass spectrometer (Thermo Scientific) as described previously [25]. LC-MS/MS files were searched against the Human Uniprot database (accessed, October 2018) using PEAKS (v8.5, BSI). Search parameters included a no-enzyme setting, precursor (10 ppm) and production (0.05 Da) tolerances, a fixed modification of carbamidomethylation on cysteine residues, and variable modifications of methionine oxidation, N-terminal pyroglutamate, N-terminal acetylation, and C-terminal amidation. The data were filtered to include only protein identifications with a 1% false discovery rate (FDR) and at least one unique peptide. The sorted cell peptidomics data have been deposited to the ProteomeXchange Consortium via the PRIDE partner repository with the data identifier PXD026621.

### 2.6 Generation of 2D monolayer cultures

Two-dimensional (2D) monolayer cultures were derived from well-established, differentiated organoids as described previously [20]. Cells were seeded onto 2% Matrigel (Corning) pre-coated 24-well plates (secretion assays), 35 mm glass-bottom dishes (MatTek, calcium imaging), or 35 mm plastic dishes (electrophysiology) and incubated at 37 °C (5% CO_2_) for 18-72 h. The cultures were washed with saline buffer (138 mM NaCl, 4.5 mM KCl, 4.2 mM NaHCO_3_, 1.2 mM NaH_2_PO_4_, 2.6 mM CaCl_2_, 1.2 mM MgCl_2_, 10 mM HEPES, 10 mM MES hydrate, 3 mM glucose; adjusted to pH 7.4 with NaOH) before the start of experiments. To evaluate the effect of acidic solutions, the pH of the standard saline buffer was adjusted with HCl.

### 2.7 Secretion assays

Test reagents were dissolved in saline buffer (200 μl/well) supplemented with fatty acid-free bovine serum albumin (0.001%). After incubation at 37 °C for 1 h, supernatants from two wells were combined into Lobind tubes (Eppendorf), centrifuged (2000 g, 5 min, 4 °C), and the resulting supernatants were snap-frozen.

Stable isotope-labeled motilin internal standard (FVPI(U^13^C_9_,^19^N-Phe)TYGE(U^13^C_6_^15^N-Leu)QRMQEKERNKGQ-acid, Cambridge Research Biochemicals) was added to each supernatant sample (50 pg) to allow relative quantification. Peptides were extracted by solid-phase extraction as described previously [25]. The eluted samples were injected onto the LC-MS/MS system immediately for analysis. Targeted analysis of peptides in the secretion samples was performed on an M-Class Acquity (Waters) microflow LC system coupled to a TQ-XS triple quadrupole mass spectrometer with an ionKey interface (Waters). The sample (10 μL) was injected onto a nanoEase M/Z Peptide BEH C18 Trap Column (130 Å, 5 μm, 300 μm × 50 mm, Waters) at 15 μL/min for a 3-min load, with mobile phases set to 90% A (0.1% formic acid in water) and 10% B (0.1% formic acid in acetonitrile). The iKey HSS T3 Separation Device (100 Å, 1.8 μm, 150 μm × 100 mm, Waters) was set at 45 °C and the analytes were separated over a 13-min gradient from 10% to 55% B, at the flow rate of 3 μL/min. The iKey was flushed for 3 min at 85% B before returning to initial conditions, resulting in an overall run time of 20 min.

Electrospray ionization was performed in the positive mode with a capillary voltage of 3 kV and a cone voltage of 30 V, collision gas flow was at 0.14 ml/min and collision energies were optimized for each transition prior to sample analysis. The selected reaction monitoring (SRM) transitions were set up based on precursor and product ion fragments for each peptide (Table S1), and each analyte was set to a dwell time of 50 ms. Data were processed on MassLynx (v 4.2, Waters). The peak area for each peptide was normalized by the peak area of motilin internal standard in each sample and expressed as fold change versus the mean of basal wells collected in parallel.

### 2.8 Electrophysiology

Perforated-patch recordings of MLN-Venus positive cells were performed as previously described [20,26]. Briefly, 2D plated organoids were washed with saline buffer and fabricated borosilicate glass electrodes (2–3 MΩ) containing internal pipette solution (76 mM K_2_PO_4_, 10 mM NaCl, 10 mM KCl, 10 mM HEPES, 1 mM MgCl_2_, 55 mM sucrose, and 10 μg/ml amphotericin B) were used to record from individual cells. Patched cells were continuously perfused with saline buffer using a gravity-fed local perfusion device before switching to a saline buffer containing test drug solutions and then switched back to saline buffer for washout. Saline buffer (described in Section 2.6) was used for all experiments except when the effect of Co_2_^+^ (2 mM) was tested. For these experiments, a modified saline buffer containing no bicarbonate and phosphate salts was used and the final concentration of NaCl was adjusted to 143 mM. Action potential spike properties were determined from short (5 ms) incremental current injections in 2 pA steps. Action potential firing rates were calculated from longer (500 ms) incremental current injections in 2 pA steps, and a threshold of 0 mV was used. The effects of low pH on the electrical activity of MLN-Venus positive cells were determined by either examining spontaneous action potential firing recorded in a current-clamp mode without injecting current (I = 0), recording current–voltage relationships with voltage ramps (1 mV/ms) applied at 5 Hz or measuring pH-change evoked currents in voltage-clamp mode whilst holding the cell at −70 mV.

### 2.9 Calcium imaging

Calcium imaging of MLN-GCaMP7s or fura2-AM-loaded MLN-Venus cultures was performed as previously described [20,27]. Briefly, live cells were imaged on an inverted microscope (Olympus IX71) every 2 s during continuous perfusion of saline buffer with or without test reagents. Mean whole-cell fura2 ratios (340/380 nm) or GCaMP7s fluorescence (488 nm) were calculated following background subtraction in Metaflour software (Molecular Devices). Responses are presented as fold change between the fura2 ratio/GCaMP7s fluorescence in test solution (maximum value recorded within 2 min of the onset of perfusion) and baseline (maximum value recorded in the 1 min prior to application of test solution).

### 2.10 Data analysis

Data are expressed as mean ± SEM throughout, with individual data points represented on graphs where appropriate. Statistical tests were performed using GraphPad Prism version 9 or DESeq2 (RNA sequencing). Statistical significance between groups was calculated using paired or unpaired t-test, Friedman test, one- or two-way analysis of variance (ANOVA), or Kruskal–Wallis test, followed by multiple comparisons tests if necessary, as indicated in the figure legends.

## 3 Results

### 3.1 Generation of motilin reporter human organoids

CRISPR-Cas9 homology donor repair was performed to generate human MLN-Venus organoids expressing the fluorescent protein downstream of the motilin coding sequence (Figure 1A–B), as previously described for human glucagon-like peptide 1 (GLP-1)-secreting L-cells [20]. Differentiated MLN-Venus organoids were fluorescence-activated cell sorted (FACSed) to obtain purified populations of Venus-fluorescent (positive) and non-fluorescent (negative) single cells (Figure 1C). qPCR analysis demonstrated a 2400-fold enrichment of *MLN* mRNA in Venus-positive cells (Figure 1D), and co-localization of MLN and Venus was further confirmed by immunohistochemistry (Figure 1E–F).

### 3.2 M-cell bulk RNA sequencing and electrical activity

**Figure 2:**
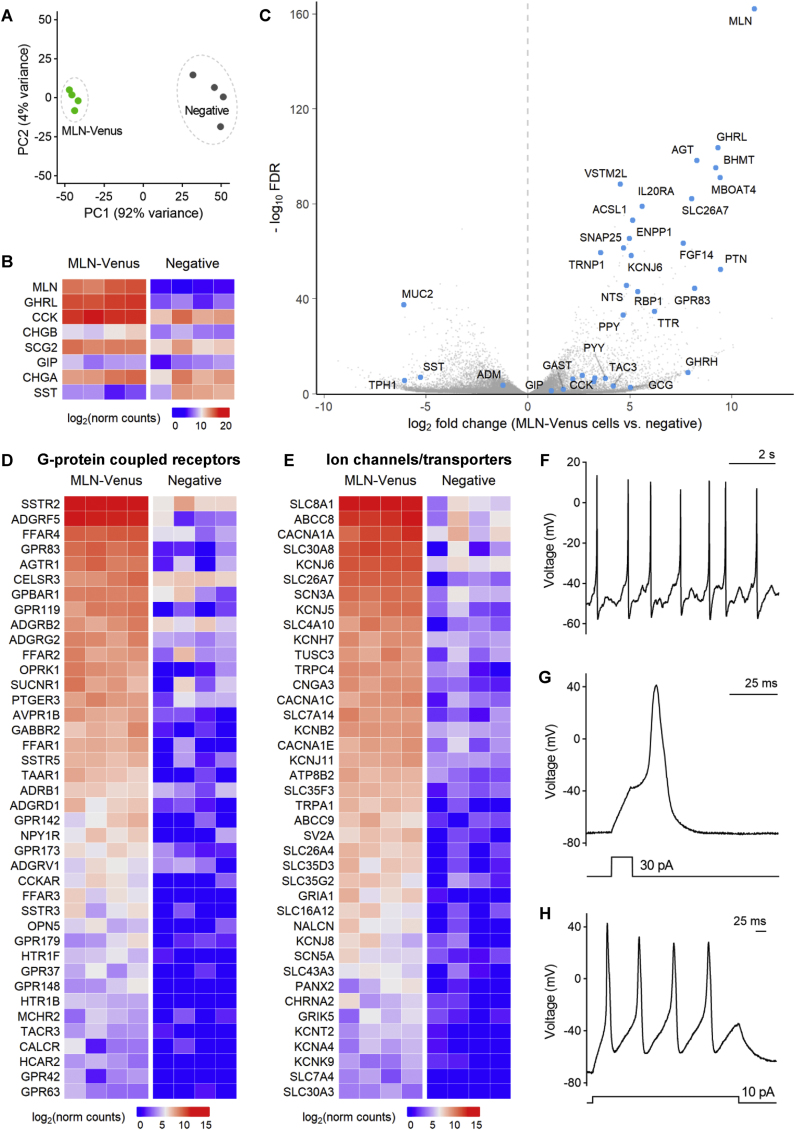
Transcriptomic analysis and electrical activity of MLN-Venus cells. **(A)** Principal component analysis comparing MLN-Venus (positive, green) and negative (grey) populations by bulk RNA sequencing, **(B)** Heatmap showing expression of selected hormones and secretory proteins in MLN-Venus and negative cells, **(C)** Volcano plot showing differential expression of selected genes in MLN-Venus cells (FDR = DESeq2 false discovery rate), **(D)** Heatmap showing expression of G-protein coupled receptors enriched in MLN-Venus cells (FDR <0.05), **(E)** Heatmap showing expression of ion channel and transporter genes enriched in MLN-Venus cells (FDR <0.05). Perforated-patch current-clamp recordings from representative MLN-Venus cells displaying, **(F)** spontaneous electrical activity at resting membrane potential, **(G)** single action potential spike triggered by a 5 ms current injection, **(H)** action potential spikes triggered by a longer (500 ms) current injection. Current injection protocols employed are shown below the individual voltage recordings.

To further characterize M-cells, we performed bulk RNA sequencing of FACS-purified MLN-Venus positive and negative (non-fluorescent) cells. The principal component analysis (PCA) demonstrated a clear separation between these two populations (Figure 2A). In addition to *MLN*, M-cells were highly enriched for the orexigenic hormone ghrelin (*GHRL*) and enzymes involved in ghrelin biosynthesis including ghrelin O-acyltransferase GOAT-*MBOAT4*) and acyl-CoA synthetase (*ACSL1*) (Figure 2B–C). Cholecystokinin (*CCK*) mRNA was also highly expressed in M-cells while levels of other duodenal hormones such as somatostatin (*SST*) and glucose-dependent insulinotropic polypeptide (*GIP*) were lower (Figure 2B). We observed enriched M-cell expression of several M-cell genes previously identified by single-cell RNA sequencing of human organoid enteroendocrine cells (EECs) [28] – angiotensinogen (*AGT*), fibroblast growth factor 14 (*FGF14*), anion exchange transporter SUT2 (*SLC26A7*), IL-20 receptor alpha (*IL2*0RA), transthyretin (*TTR*) and TMF-regulated nuclear protein 1 (*TRNP1*) (Figure 2C).

Several G-protein coupled receptors (GPCRs) were highly enriched in M-cells and may therefore contribute to the regulation of MTN release (Figure 2D). Several GPCRs known to be involved in postprandial nutrient sensing in EECs were expressed at high levels [[29], [30], [31]]. These included receptors for triglyceride digestion products (*FFAR1/4*, *GPR119*), short-chain fatty acids (*FFAR2/3*), bile acids (*GPBAR1*)*,* amino acids (*GPR142*), and trace amines (*TAAR1*). M-cells also expressed receptors for several neurohormonal signals, including several peptide hormones previously implicated in the control of metabolism (*SSTRs*, *CCKAR*, *NPY1R, AGTR1*, *AVPR1B*), less well-established peptides (*GPR83* [pro-SAAS/PEN-receptor] [32]; *GPR173* [Phoenixin-receptor] [33]) and small-molecule neurotransmitters (*GABBR2, OPRK1*). Several other neurotransmitter receptors showed expression in M-cells that might underlie modulation by vagal and/or enteric neurons (Figure S1A). M-cells expressed voltage-gated ion channels previously identified in other electrically excitable EECs (Figure 2E), including Na_v_1.3 (*SCN3A*)*,* P/Q-type Ca_v_2.1 (*CACNA1A*)*,* L-type Ca_v_1.2 (*CACNA1C*)*,* K_v_2.2 (*KCNB2*) and inward-rectifying channels K_ir_3.2/3.4/6.2 (*KCNJ6/5/11*). Consistent with the expression of voltage-gated ion channels involved in action potential generation and similar to other enteroendocrine cells [20], M-cells are electrically active. At resting membrane potential (mean = −54.6 ± 3.1 mV), perforated-patch recordings on Venus-fluorescent M-cells plated in 2D cultures showed spontaneous firing of action potentials in 9/10 cells, while evoked action potentials were observed in all 11 cells following current injection (Figure 2F–H). The threshold for M-cell action potential firing was −34.3 ± 1.7 mV and the peak depolarisation was +29.7 ± 2.9 mV, properties similar to previously characterised human ileal L-cells [20].

### 3.3 LC-MS/MS analysis of MLN-Venus sorted cells and secreted peptides

**Figure 3:**
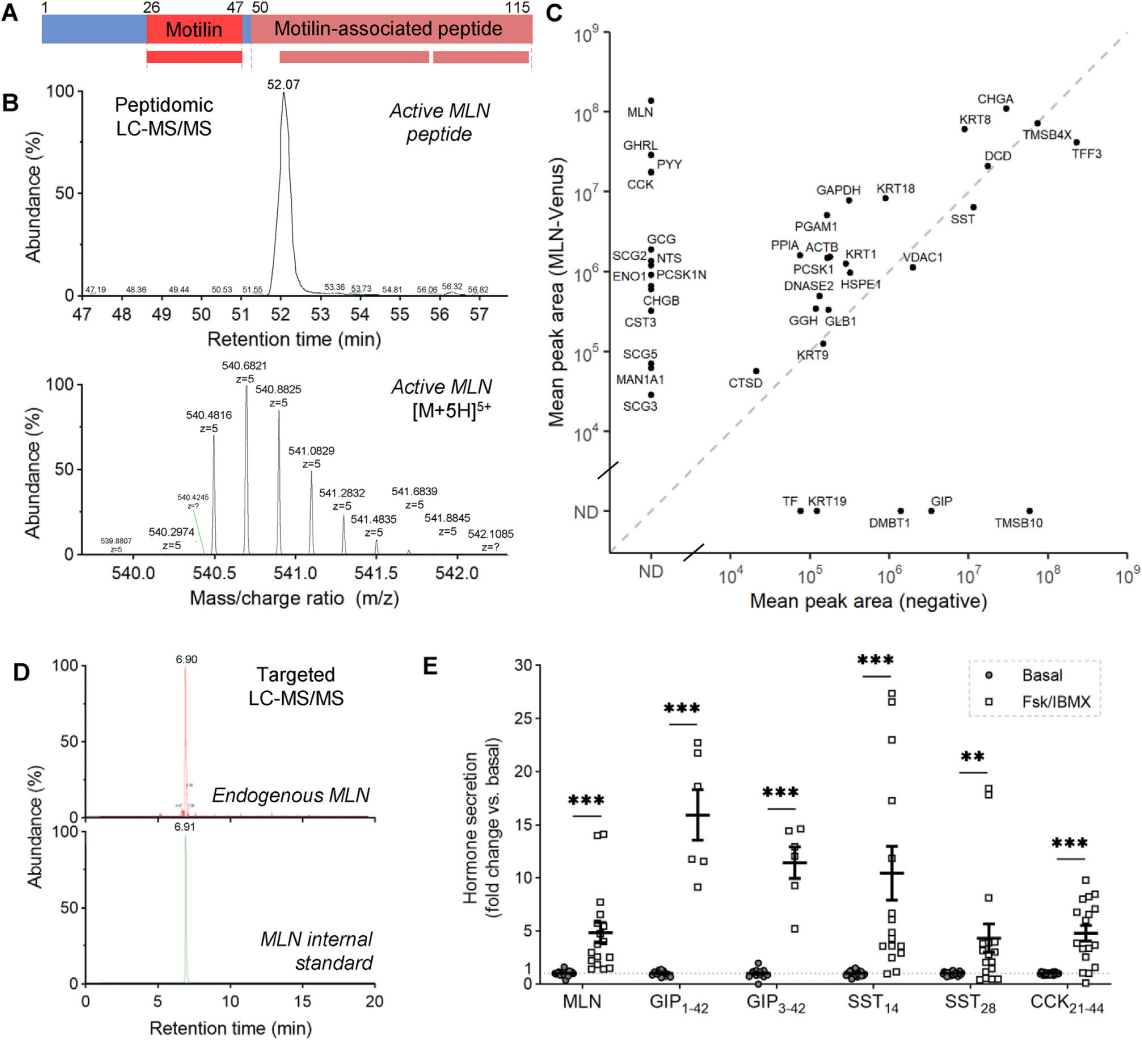
LC-MS/MS analysis of MLN-Venus sorted cells and secretion supernatants. **(A)** Most abundant processed forms of motilin (MLN_26-47_) and motilin-associated peptide (MLN_57-91_ and Pyr^1^-MLN_93-113/114_) detected in MLN-Venus sorted cells by LC-MS/MS, **(B)** Extracted ion chromatogram showing retention time and mass/charge ratio (*m/z*) for the active 22-amino acid motilin peptide ([M+5H]^5+^ ion shown, along with ^13^C isotopes), **(C)** LC-MS/MS peptidomics of FACSed MLN-Venus cells, compared with negative non-fluorescent cells (peptides detected are combined per parental protein, labeled by gene name and expressed as mean peak area). ND = not detectable. **(D)** Targeted analysis of MLN in secretion supernatants, using stable isotope-labeled internal standard to enable relative quantification, **(E)** Peptide secretion in response to 10 μM forskolin (fsk) plus 100 μM isobutylmethylxanthine (IBMX), measured in supernatants by targeted LC-MS/MS and expressed as fold change versus basal condition measured in parallel. ∗∗p < 0.01, ∗∗∗p < 0.001 by Mann–Whitney test, mean ± SEM presented (n = 12–20 wells from 6 to 11 independent experiments).

We next examined the peptide content of MLN-Venus sorted cells by mass spectrometry. Using a peptidomics approach, we reliably detected the fully processed 22-amino acid active motilin peptide (Figure 3A–C), as well as 2 peptides from motilin-associated peptides located in the C-terminal region of proMLN (Figure 3A; S1). Consistent with the transcriptomic data, M-cells also produced octanoylated acyl-GHRL_28_, CCK_21-44_, a reliably detectable proCCK fragment co-produced with active CCK (itself difficult to detect with the same instrument settings), PYY and proglucagon-derived peptides, including GLP-1, while mature forms of GIP and SST were found at higher levels in the MLN-Venus negative population (Figure 3C; S2).

We developed a high throughput LC-MS/MS assay to measure motilin secretion from stimulated 2D organoid-derived cultures, as well as other prespecified peptides. The inclusion of a stable isotope-labeled motilin internal standard enabled relative quantification of motilin levels in secretion supernatants (Figure 3D). Using a mass spectrometry approach helped measure other duodenal hormones in parallel, as shown for the cyclic AMP-dependent secretion of GIP_1-42_, GIP_3-42_, SST_14_, SST_28_, and CCK_21-44_ in response to forskolin plus isobutylmethylxanthine (Figure 3E).

### 3.4 Motilin secretion is stimulated via GPBAR1-and FFA1-dependent pathways

**Figure 4:**
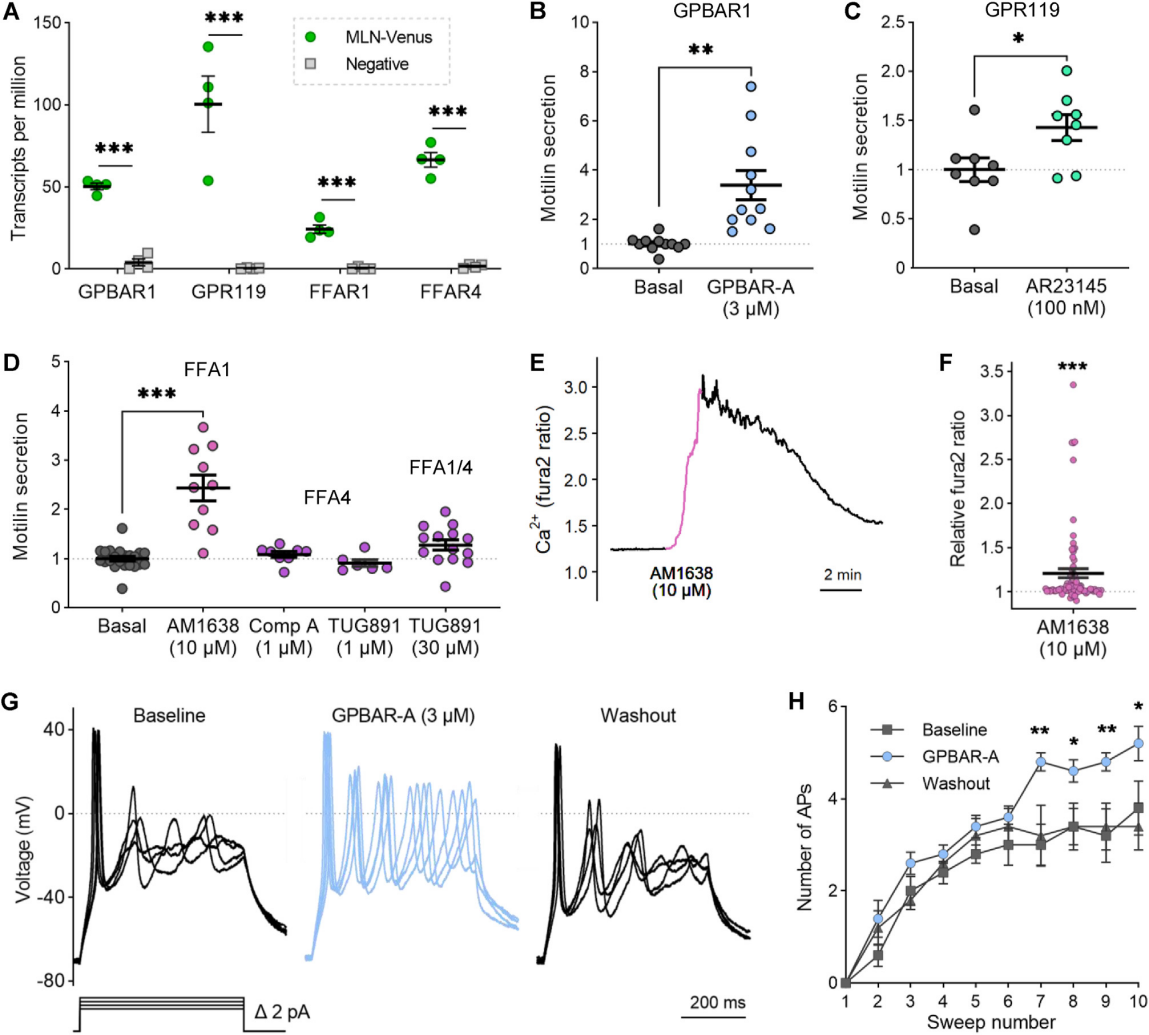
GPBAR1, FFA1, and GPR119 agonists induce motilin secretion. **(A)** Bulk RNA sequencing data showing transcripts per million (TPM) of fat and bile sensing receptors in MLN-Venus and negative cells, **(B**–**D)** Motilin secretion in response to indicated agonists (expressed as fold change versus basal condition measured in parallel). ∗p < 0.05, ∗∗p < 0.01, ∗∗∗p < 0.001 by Welch's t-test, or Browne-Forsyth and Welch ANOVA with Dunnett's multiple comparisons (n = 6–14 wells from 3 to 7 independent experiments), **(E)** Fura2 (340/380 nm) ratio in one representative MLN-Venus cell during perfusion of FFA1 agonist AM1638 (10 μM, pink), **(F)** Calcium signal across several cells, measured as fold change in maximal fura2 ratio, as in (E). ∗∗∗p < 0.001 by one-sample test (n = 77 cells from 7 independent experiments), **(G)** Action potentials evoked by incremental (+2 pA) 500 ms current steps from 12 to 18 pA in the presence (blue) or absence (black) of GPBAR-A (3 μM). The dotted line at 0 mV represents the threshold used to count action potential spikes. The same axis scale is applied across all traces, and the current injection protocol applied is shown below voltage traces, **(H)** The number of action potentials recorded as in (G). ∗p < 0.05, ∗∗p < 0.01 by two-way ANOVA with Sidak's multiple comparisons (n = 5 cells). Mean ± SEM presented.

Since fat ingestion and gallbladder emptying stimulate motilin release in humans [[16], [17], [18]], we investigated the molecular mechanisms underlying M-cell sensing of bile and products of lipid digestion. Our RNA sequencing data demonstrated enriched expression of the G-protein bile acid receptor 1 (*GPBAR1)*, long-chain fatty acid receptors, namely *FFAR1* and *FFAR4* (also known as FFA1/GPR40 and FFA4/GPR120, respectively), and the monoacylglycerol receptor *GPR119* (Figure 4A). As the endogenous ligands for these receptors are non-specific, we used selective synthetic ligands to study their function. Motilin secretion was significantly stimulated by the GPBAR1-agonist GPBAR-A (3 μM, 3.4-fold), the GPR119 agonist AR231453 (100 nM, 1.5-fold), and the FFA1 agonist AM1638 (10 μM, 2.4-fold) (Figure 4B–D). Even though there was a trend towards stimulated motilin secretion with the FFA4 agonist TUG891 at the higher concentration (30 μM) tested (1.3-fold; p = 0.06), at this concentration it is known to also agonise FFA1 [34]. Neither a lower concentration of TUG891 (1 μM), which should not activate FFA1, nor the FFA4-selective compound A [35] stimulated motilin secretion (Figure 4D). This supports long-chain fatty acid signalling via FFA1 as a potent stimulus for motilin release.

To gain further mechanistic insight into FFA1-and GPBAR1-dependent stimulation of motilin release, we measured intracellular calcium levels (after loading cells with fura2) or electrical activity of MLN-Venus cells in response to agonist application. Activation of the G_q_-coupled receptor FFA1 with AM1638 increased the calcium-dependent fura2 fluorescence ratio by >15% in 19/77 MLN-Venus cells (Figure 4E–F). Stimulation of the G_s_-coupled receptor GPBAR1 significantly increased the evoked action potential firing rate in MLN-Venus cells (Figure 4G–H). A similar effect on evoked action potential firing rate was described for human ileal L-cells [20], which suggests a common mechanism of action of GPBAR1 on these two enteroendocrine cell types.

### 3.5 Motilin secretion is stimulated by acidification

**Figure 5:**
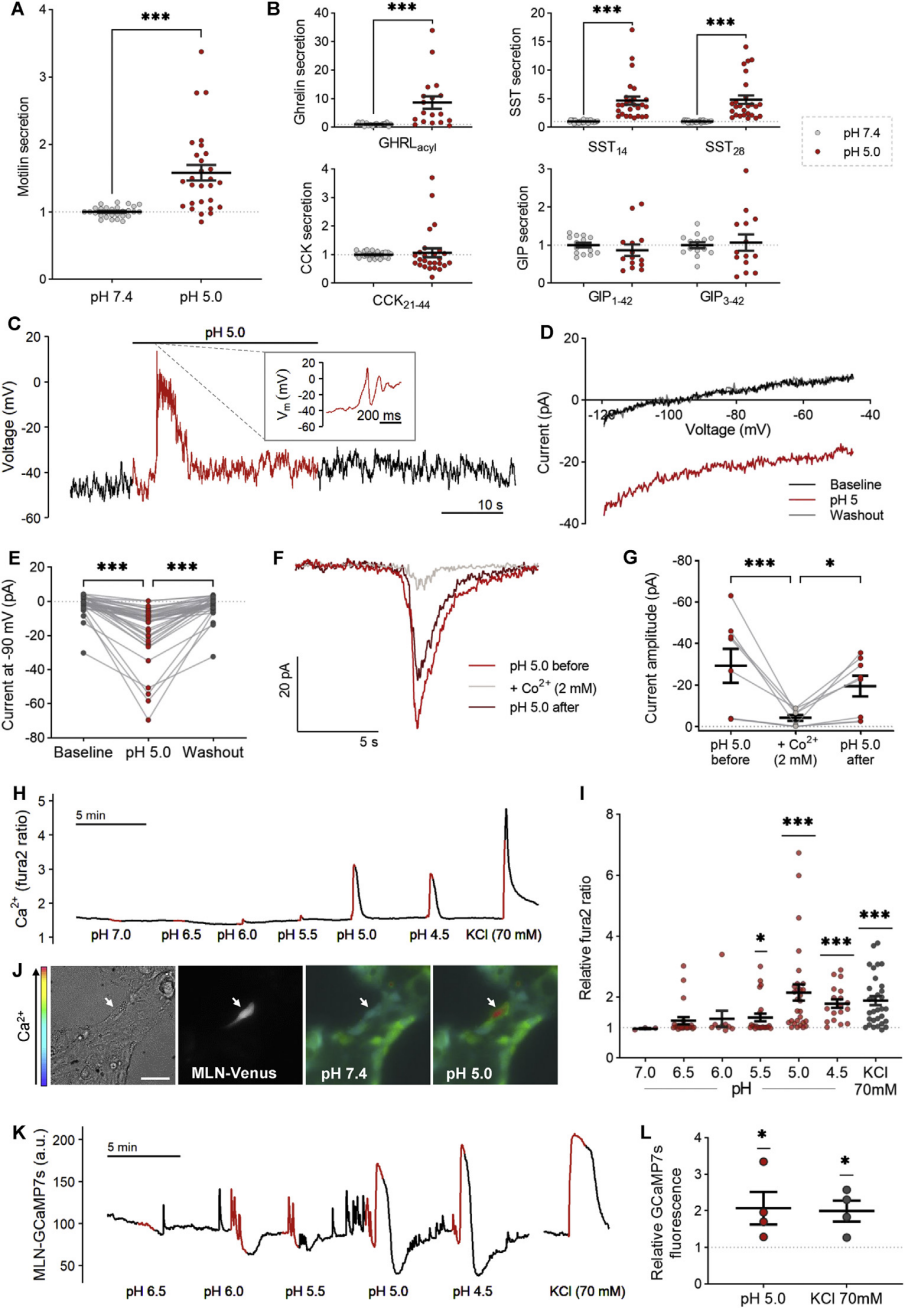
Stimulation of motilin secretion by acidification. **(A)** Motilin secretion from human organoid-derived 2D cultures incubated with pH 5.0 saline, expressed relative to basal (pH 7.4) wells measured in parallel, ∗∗∗p < 0.001 by unpaired t-test (n = 28 wells from 14 independent experiments). **(B)** As in (A) for other peptide hormones. **(C)** Recording of membrane voltage at the resting membrane potential of one representative MLN-Venus cell during perfusion with pH 7.4 (black) or pH 5.0 (red) saline. **(D)** Current–voltage relationship measured during voltage ramps of 1 mV/ms, showing induction of an inward current during perfusion with pH 5.0 saline (red) compared to pH 7.4 (black/grey) in one representative MLN-Venus cell. **(E)** Current measured at −90 mV from multiple cells, recorded as in (D). ∗∗∗p < 0.001 by Friedman test with Dunn's multiple comparisons (n = 36 cells). **(F)** Current recording in one representative MLN-Venus cell clamped at −70 mV, in response to pH 5 saline with (grey) or without (red) 2 mM Co^2+^. **(G)** Peak current amplitude from multiple cells recorded as in (F). ∗p < 0.05, ∗∗∗p < 0.001 by Friedman test with Dunn's multiple comparisons (n = 8 cells) **(H)** Fura2 (340/380 nm) ratio in one representative MLN-Venus cell during perfusion of increasingly acidic saline solutions (red). **(I)** Data from multiple cells recorded as in (H), as fold change in maximal fura2 ratio. ∗p < 0.05, ∗∗∗p < 0.001 by one-sample t-tests (n = 3–29 cells from 1 to 4 independent experiments). **(J)** Calcium elevation in an MLN-Venus cell (brightfield left, Venus excitation middle left) in response to application of pH 5.0 saline, as measured by a change in Fura2 excitation ratio (right). Scale bar 50 μm. **(K)** GCaMP7s fluorescence (488 nm excitation) in arbitrary units (a.u.) in one representative MLN-GCaMP7s cell during perfusion of increasingly acidic saline solutions (red). **(L)** Data from multiple cells recorded as in (K), as fold change in maximal GCaMP7s fluorescence. ∗p < 0.05 by one-sample t-tests (n = 4 cells from 3 independent experiments). Mean ± SEM presented.

Duodenal acidification evokes motilin release both *in vivo* [13] and *in vitro* [17], leading us to hypothesise that low pH will activate human M-cells. Motilin secretion from organoid-derived cultures was modestly but significantly stimulated following 1-h incubation at pH 5.0, (1.6-fold; Figure 5A). We also observed secretion of acyl-ghrelin and both active forms of somatostatin (SST_14_/SST_28_), but not GIP or CCK_21-44_, in response to low pH (Figure 5B).

During electrophysiological recordings of membrane voltage in MLN-Venus cells at resting membrane potential, application of saline buffer at pH 5.0 induced a transient membrane depolarisation (mean depolarisation = +34.6 ± 5.4 mV), which triggered action potential firing in 7/9 cells (Figure 5C). Further examination of the current–voltage relationship of the current activated by low pH revealed a transient inward current was responsible for the depolarisation observed (Figure 5D–E), which was confirmed with continuous voltage-clamp recordings (Figure 5F). This inward current activated by low pH was blocked by the presence of extracellular Co^2+^ and therefore likely mediated by influx of Na ^+^ or Ca^2+^ (Figure 5F–G). Similarly, perfusion of increasingly acidic solutions evoked calcium elevations in fura2-loaded M-cells (Figure 5H–I). The calcium response to pH 5.0 was selective to MLN-Venus cells and a small proportion of non-fluorescent cells (likely another enteroendocrine cell type), and so this pH, which is within the physiologically observed range in the proximal duodenum [36,37], was used for further experiments (Figure 5J). To further validate that this effect was not a result of the pH sensitivity of fura2, we generated an MLN-GCaMP7s reporter line. GCaMP7s fluorescence increased upon exposure to pH 5.0, confirming the elevation in intracellular calcium (Figure 5K-L). By contrast, alkaline solutions did not evoke significant increases in M-cell fura2 ratio or stimulation of motilin secretion (Figure S3A–C).

We examined our RNA sequencing dataset to identify potential pH-sensing mechanisms [38]. We found highly enriched expression in MLN-Venus cells of the acid-sensitive transient receptor potential channel *TRPC4* [39] but inhibition of TRPC4 with ML204 did not alter motilin secretion (Figure S4A–B). The two-pore potassium channels TALK1/*KCNK16* and TASK1/*KCNK3* were also enriched in M-cells (Figure S4C), but would not account for the observed pH-dependent inward current, while known proton-sensing GPCRs were largely undetectable (Figure S4D).

**Figure 6:**
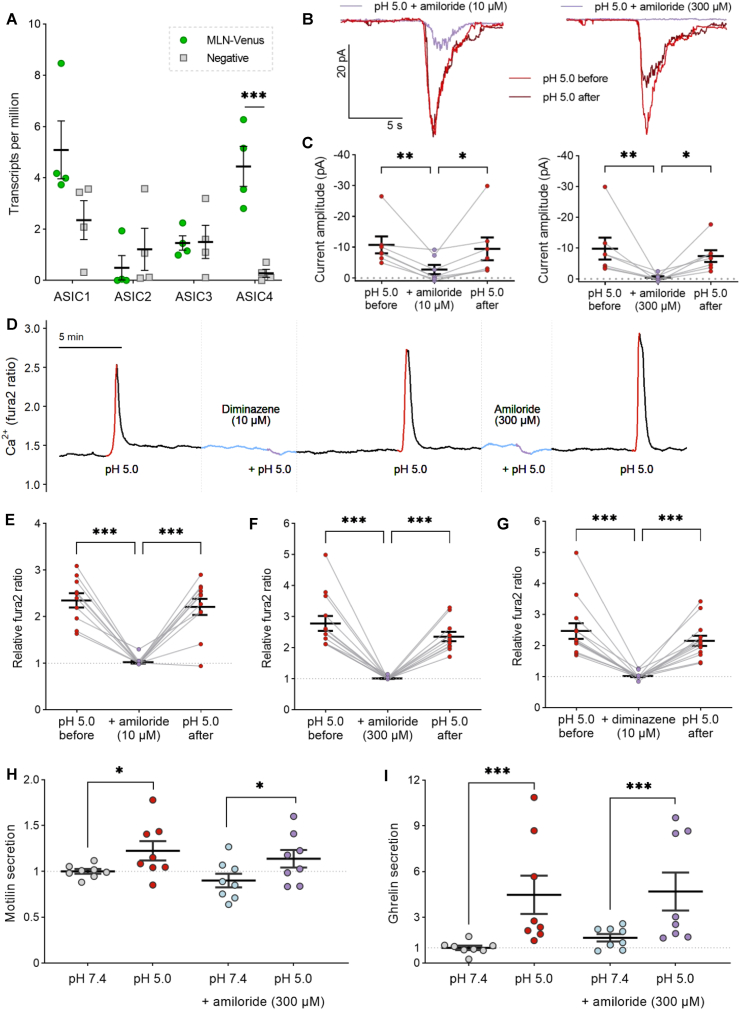
Acute responses to low pH are mediated by acid-sensing ion channels (ASICs). **(A)** Bulk RNA sequencing data showing transcripts per million (TPM) of acid-sensing ion channels in MLN-Venus and negative cells. **(B)** Current recordings from a representative MLN-Venus cell held at −70 mV in response to pH 5 saline with (purple) or without (red) the ASIC inhibitor amiloride (10 μM left, 300 μM right). **(C)** Peak current amplitude from multiple cells, as measured in (B). ∗p < 0.05, ∗∗p < 0.01 by Friedman test with Dunn's multiple comparisons (n = 7 cells). **(D)** Fura2 (340/380 nm) ratio in one representative MLN-Venus cell during perfusion of pH 5.0 saline alone (red) or in the presence of ASIC inhibitors (purple), amiloride (300 μM) and diminazene (10 μM). **(E**–**G)** Data from multiple cells recorded as in (D), as fold change in maximal Fura2 ratio. ∗∗∗p < 0.001 by repeated measures one-way ANOVA with Dunnett's multiple comparisons (n = 11–13 cells from 2 independent experiments). **(H–I)** Secretion of motilin (H) and acyl-ghrelin (I) in response to pH 5.0 in the presence or absence of amiloride (300 μM). ∗p < 0.05, ∗∗∗p < 0.001 by two-way ANOVA with Sidak's multiple comparisons (n = 8 wells from 4 independent experiments). Mean ± SEM presented.

Several acid-sensing ion channels (*ASIC1-4*) were expressed in MLN-Venus cells (Figure 6A). Amiloride – a non-selective inhibitor of ASICs, in addition to other channels such as the epithelial sodium channel (ENaC) – blocked low pH-induced currents at both low (10 μM) and high (300 μM) concentrations (Figure 6B–C). This inhibition was also observed in calcium imaging (Figure 6D–F). To specifically target ASICs, we used the antiprotozoal drug diminazene, which does not inhibit ENaC [40], and observed that this drug also ablated low pH-induced calcium transients (Figure 6D/G). However, amiloride did not affect the acid-evoked secretion of motilin (Figure 6H) or acyl-ghrelin (Figure 6I), which is likely released from the same cell population. This indicates that while ASICs mediate a transient pH-sensitive current that increases M-cell excitability and elevates intracellular calcium levels, which are important for acute M-cell responses to low pH, other mechanisms may be involved in mediating prolonged MLN secretion.

### 3.6 Cholinergic and adrenergic stimulation does not affect motilin secretion

As acetylcholine stimulates motilin release from canine duodenum [41], and the muscarinic acetylcholine receptors M3 (*CHRM3*, Gq-coupled) and M4 (*CHRM4*, Gi-coupled) were expressed in M-cells (Figure S1A), we assessed the effects of cholinergic stimulation. In response to Carbachol (100 μM), a calcium response was observed in 15/22 MLN-Venus cells (Figure S1B) but there was no effect on motilin secretion (Figure S1C). Similarly, low levels of several adrenoreceptors were expressed in M-cells (Figure S1A), including the Gi-coupled α_2A_ (*ADRA2A)* and Gs-coupled β_1_ (*ADRB1)*. Noradrenaline (30 μM) stimulated calcium elevations in 5/13 M-cells; however, they induced no motilin secretion (Figure S1B–C).

## 4 Discussion

The lack of motilin expression in rodent models or any existing human cell line has prevented the detailed study of motilin secretion mechanisms to date. Here we established a human duodenal organoid model with fluorescently labeled M-cells, which enabled the identification and purification of motilin-expressing cells for transcriptomic, peptidomic, and functional characterization. Combined with the development of an efficient targeted LC-MS/MS-based assay, we could demonstrate GPBAR1-, FFA1-and low pH-dependent stimulation of motilin release from human cells *in vitro*.

The orexigenic hormone ghrelin was highly enriched in duodenal organoid M-cells, as previously demonstrated in human and pig tissue [42]. We detected the active acyl-ghrelin in secretion supernatants and sorted M-cells, suggesting that small intestinal EECs may contribute to circulating ghrelin levels. This strengthens human tissue peptidomics and reports that some plasma ghrelin (∼40%) is maintained following total gastrectomy [[43], [44], [45]]. Our RNA sequencing and peptidomics of sorted cells also suggested some co-localization of MLN with CCK-expressing EECs, but not SST and GIP. The transcriptome of M-cells has recently been assessed by single-cell RNA sequencing of EECs from *NEUROG3*-overexpressing human organoids [28]. Our bulk RNA sequencing data confirmed enrichment of many previously identified M-cell genes (e.g., *AGT*, *FGF14*, *SLC26A7*, *IL2*0RA, *TTR,* and *TRNP1*), while providing a higher read depth and thus improving detection of several lowly expressed genes critical for cell signaling, predominantly GPCRs and ion channels. To our knowledge, the current study is first to demonstrate that M-cells are electrically active. The profile of ion channel expression and action potential properties were similar to human ileal L-cells previously described [20]. M-cells expressed receptors for a range of nutrient and neurohormonal signals which may be important in regulating motilin release. We assessed the functions of a subset of these receptors, namely those involved in sensing products of fat digestion, bile acids, and luminal pH.

Several *in vivo* studies have previously shown that oral ingestion of a fat-rich meal or intravenous lipid emulsion infusion stimulates motilin release [[16], [17], [18]], but the molecular mechanisms remained undefined. Here we demonstrated that activation of both the long-chain fatty acid receptor (FFA1) and the monoacylglycerol receptor (GPR119) stimulated significant motilin secretion from organoid cultures. We also showed that in around a quarter of duodenal M-cells, the FFA1 agonist AM1638 evokes calcium responses. The other long-chain fatty acid receptor, FFA4, was also enriched in the MLN-Venus population and has been linked to inhibition of gastric ghrelin-secreting cells in mice [46]; however, agonists of this receptor did not significantly affect motilin release *in vitro*. Normal postprandial motilin levels appear to depend on a meal's nutritional makeup, with existing studies reporting an increase, decrease, or no effect of mixed meal ingestion on plasma motilin levels [8]. In this model, we did not investigate any stimuli such as glucose - which are predicted to inhibit motilin release, but this will form an important component of future work.

A selective synthetic agonist (GPBAR-A) of the G-protein bile acid receptor GPBAR1, which was highly expressed and enriched in M-cells, also strongly stimulated motilin secretion. GPBAR-A increased evoked action potential firing in M-cells, similar to our previous observations of human and mouse ileal GLP-1-secreting L-cells [20,47]. The release of bile from the gallbladder into the duodenum has causally been linked to motilin secretion in humans [14,48] and the bile acid taurocholate stimulates motilin release from perifused duodenal tissue pieces *in vitro* [17]. This suggests GPBAR1 underlies the bile acid-induced stimulation of motilin secretion, which may be relevant both postprandially and during fasting. However, as maximal gallbladder emptying occurs during the strong contractions of phase III of the MMC, bile acids are unlikely to be responsible for the motilin elevations during the MMC, which peak prior to induction of phase III [3].

It has previously been demonstrated that duodenal instillation or *in vitro* perfusion of human duodenal mucosal pieces with hydrochloric acid (pH 1–2) strongly stimulates motilin secretion [13,17,49]. Moreover, we showed that proton-gated ASICs were partially responsible for M-cell stimulation in response to low pH, as inhibition with amiloride blocked acid-induced depolarisation, and both amiloride and the selective ASIC inhibitor diminazene ablated acid-induced calcium elevations. Although ASICs have primarily been investigated for their roles in nociception [38], several examples of non-neuronal ASIC expression have been documented [50], including one study which implicated ASIC1a in bicarbonate secretion from duodenal epithelial cells [51].

Despite the clear effect of ASIC inhibitors on acute single-cell responses measured by electrophysiology or Ca^2+^ imaging, low pH-evoked secretion of motilin and ghrelin during a longer 1-h incubation was relatively modest and not affected by amiloride. Therefore, additional mechanisms may be involved over an extended period, for example, proton-sensing GPCRs or cross-talk with other cell populations. However, we could not observe an enrichment of known proton-sensing GPCRs in the M-cell transcriptome, and D-cell derived SST, which would be expected to inhibit rather than stimulate motilin release [52], was also elevated by low pH. Between meals, motilin is released during phase II of the MMC, which is associated with a drop in duodenal pH due to the entry of acidic stomach content [36,37]. pH measured at the duodenal bulb throughout phase II is highly variable amongst individual subjects and rapidly fluctuates within a range of 2.0–7.5 [37]. Although we have investigated the effect of prolonged incubation at low pH, M-cells may be more responsive to acute changes in pH, since there is also a steep pH gradient along the proximal duodenum [36,53]. In the Asian house shrew (*Suncus murinus*), secondary duodenal alkalinization in response to acidification-induced serotonin release has been proposed to underlie motilin secretion [54]; however, we could not establish a direct effect of high pH on human M-cells, suggesting a species difference. Motilin release during phase II may also be further mediated by intestinal contractions or the autonomic nervous system. We did not assess the effects of mechanical stimulation in this study, but neither cholinergic nor adrenergic activation evoked motilin release.

In addition to motilin, secretion of other duodenal hormones by acidification has also been described including secretin [55], somatostatin [56,57], and the neuronal vasoactive intestinal peptide [58]. This is generally downstream of increased gastric emptying but, to our knowledge, no molecular mechanisms have yet been proposed and future studies should therefore investigate whether ASICs also play a role in acid-sensing in other enteroendocrine populations.

## 5 Conclusion

In this study, we performed the first extensive functional characterization of human motilin-expressing cells *in vitro*. Our recently optimized protocols for the generation and labeling of EECs in human organoid culture were readily extendable to a different cell type and intestinal region, enabling identification of M-cells for electrophysiology, calcium imaging, and FACS purification [20]. Motilin secretion was induced by activation of the bile acid receptor GPBAR1 and receptors sensing products of fat digestion, FFA1, and GPR119. Low pH also stimulated duodenal M-cells, an effect mediated by acid-sensing ion channels. These cellular mechanisms suggest several important pathways for the physiological control of motilin secretion during the interdigestive MMC and in the postprandial state.
