# Supplementary material for: Stimulation of motilin secretion by bile, free fatty acids, and acidification in human duodenal organoids
Source: Mol Metab. 2021 Oct 15;54:101356. doi: 10.1016/j.molmet.2021.101356 (PMC8590067; doi:10.1016/j.molmet.2021.101356)
Supplement: Supplementary file 1 — Multimedia component 1 [file mmc1.pdf]

Analyte	Precursor ion (m/z)	Product ion (m/z)	Collision energy (eV)	Retention time (min)
MLN	540.6	748.37	18	6.95
GIP_{1-42}	831.6	207.05	27	8.06
GIP_{3-42}	792.4	841.03	20	7.91
SST_{14}	546.6	726.30	15	6.70
SST_{28}	630.7	748.50	18	6.64
CCK_{21-44}	842.1	612.99	33	5.77
$GHRL_{acyl}$	562.6	689.05	18	6.12
MLN internal standard	544.6	613.50	18	6.95

Table S1: Selected reaction monitoring transitions for peptides measured by targeted LC-MS/MS

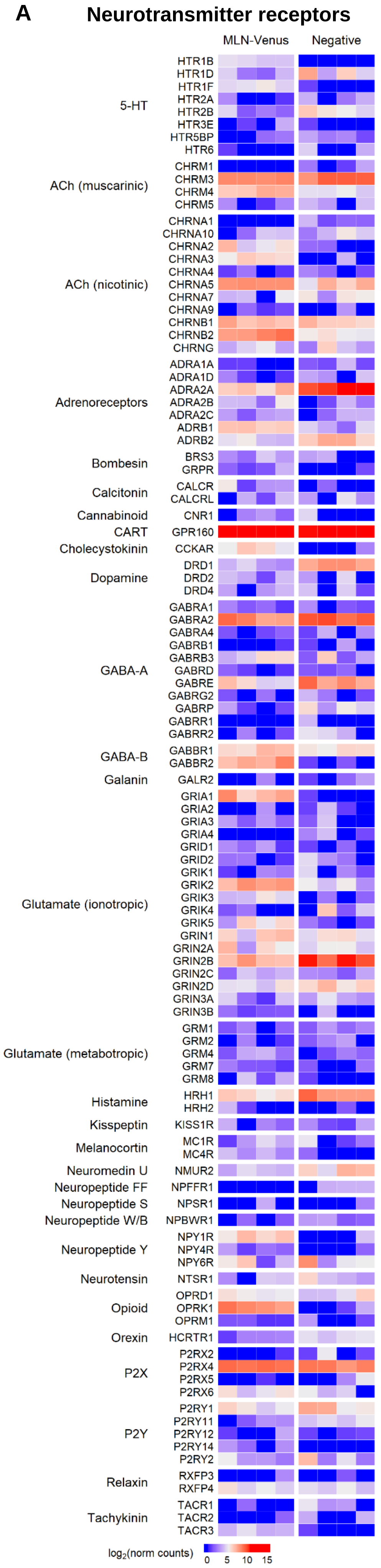

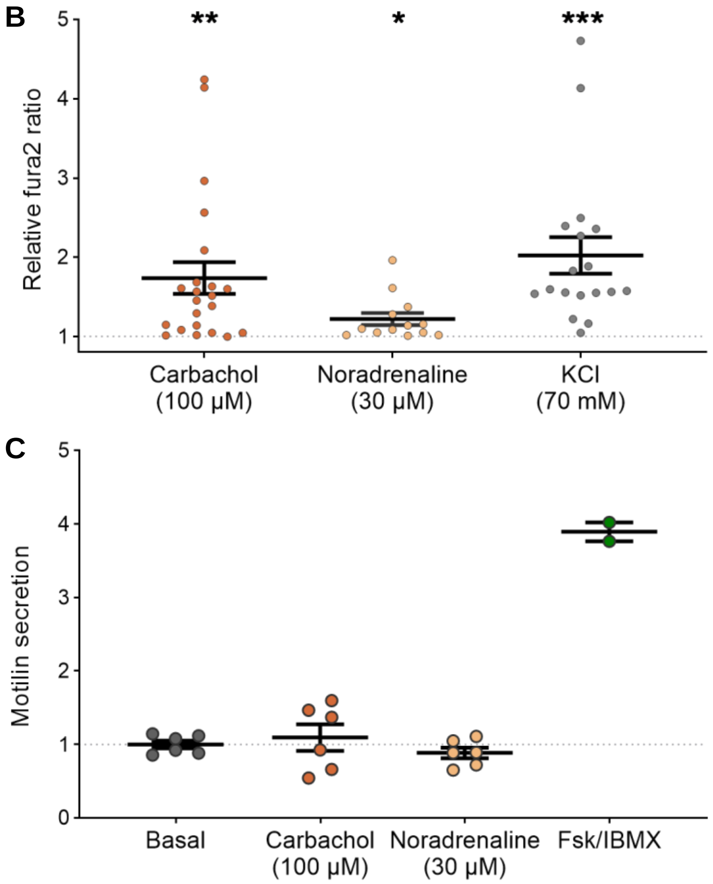

Figure S1 – Neurotransmitter receptor expression and effects of cholinergic or adrenergic stimulation

(A) Heatmap showing expression of neurotransmitter receptor genes in MLN-Venus and negative cells.
(B) Calcium signal across several cells in response to carbachol (100 µM) or noradrenaline (30 µM), measured as fold change in maximal fura2 ratio. * $p < 0.05$, ** $p < 0.01$, *** $p < 0.001$ by one sample test (n = 13-22 cells from 2-3 independent experiments).
(C) Motilin secretion in response to carbachol or noradrenaline (expressed as fold change versus basal condition measured in parallel). Non-significant by Browne-Forsyth and Welch ANOVA with Dunnett's multiple comparisons (n = 6 wells from 3 independent experiments).

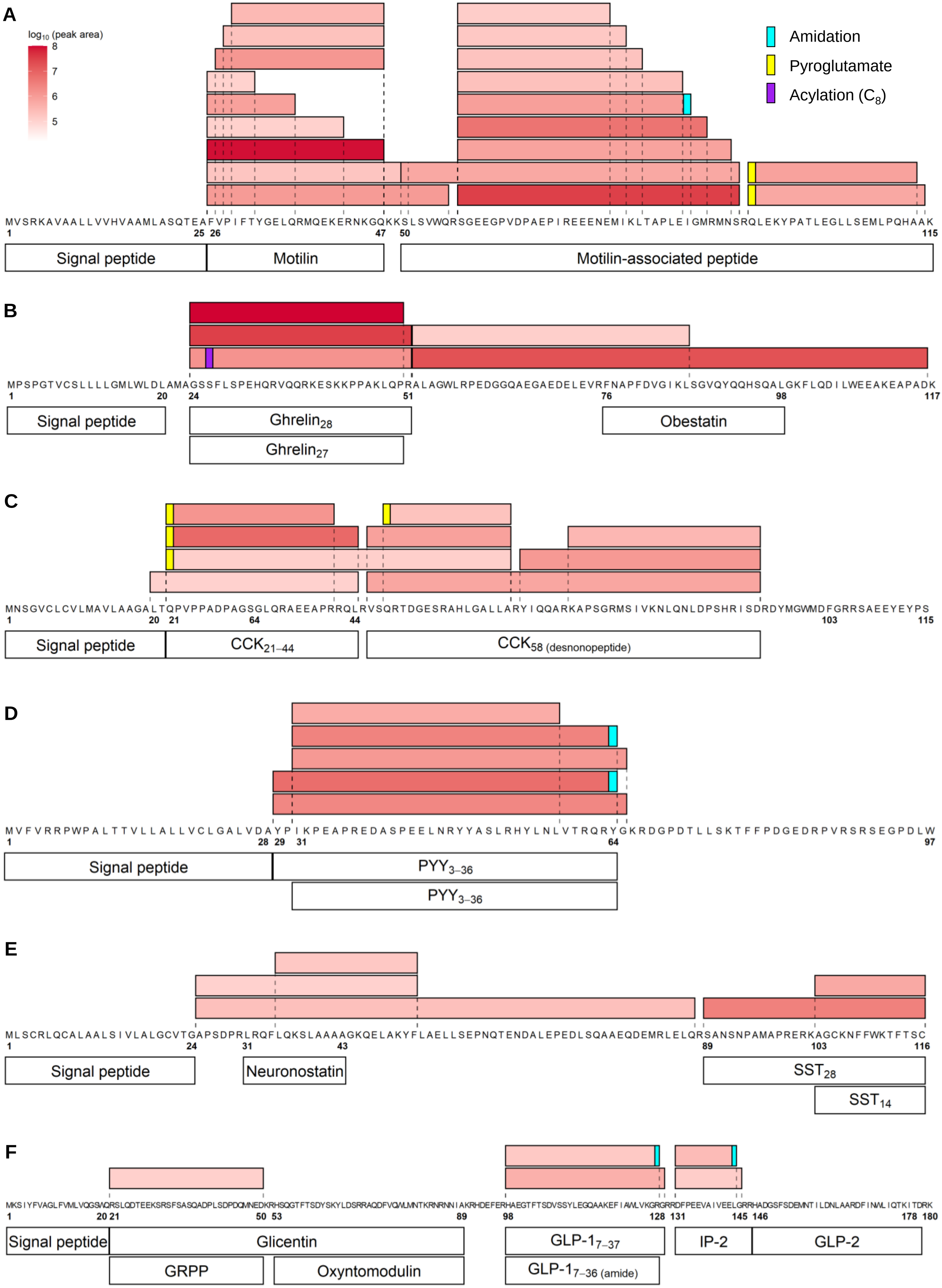

A
log10 (peak area)
8
7
6
5
Amidation
Pyroglutamate
Acylation (C8)
MVSRKAVAALLVVHVAAMLASQTEAFVPIFTYGELQRMQEKERNKGQKKSLSVWQRSGEEGPVDPAEPIREEENEMIKLTAPLEIGMRMNSRQLEKYPATLEGLLSEMLPQHAAK
1 25 26 47 50 115
Signal peptide
Motilin
Motilin-associated peptide
B
MPSPGTVCSLLLLGMLWLDLAMAGSSFLSPEHQRVQQRKESKKPPAKLQPRALAGWLRPEDGGQAEGAEDELEVRFNAPFDVGIKLSGVQYQQHSQALGKFLQDILWEEAKEAPADK
1 20 24 51 76 98 117
Signal peptide
Ghrelin28
Ghrelin27
Obestatin
C
MNSGVCLCVLMAVLAAGALTQPVPPADPAGSGLQRAEEAPRRQLRVSQRTDGESRAHLGALLARYIQQARKAPSGRMSIVKNLQNLDPSHRISDRDYMGWMDFGRRSAEEYEYPS
1 20 21 64 44 103 115
Signal peptide
CCK21–44
CCK58 (desnonopeptide)
D
MVFVRRPWPALTTVLLALLVCLGALVDAYPIKPEAPREDASPEELNRYYASLRHYLNLVTRQRYGKRDGPDTLLSKTFFPDGEDRPVRSRSEGPDLW
1 28 29 31 64 97
Signal peptide
PYY3–36
PYY3–36
E
MLSCRLQCALAALSIVLALGCVTGAPSDPRLRQFLQKSLAAAAGKQELAKYFLAELLSEPNQTENDALEPEDLSQAAEQDEMRLELQRSANSNPAMAPRERKAGCKNFFWKTFTSC
1 24 31 43 89 103 116
Signal peptide
Neuronostatin
SST28
SST14
F
1 20 21 50 53 89 98 128 131 145 146 178 180
Signal peptide
Glicentin
GRPP
Oxyntomodulin
GLP-17–37
GLP-17–36 (amide)
IP-2
GLP-2

Figure S2 (*previous page*) – Processed gut hormone peptides detected in MLN-Venus sorted cells

Peptides from motilin (**A**), ghrelin (**B**), cholecystokinin/CCK (**C**), peptide YY/PYY (**D**), somatostatin/SST (**E**) and proglucagon (**F**) prohormones detected by peptidomic liquid chromatography-mass spectrometry (LC-MS/MS) in MLN-Venus sorted cells. Peptides detected in at least 2 1 out of 3 samples are shown, colour-coded according to mean peak area (dark red represents highest abundance). Peak areas for ghrelin-27, ghrelin-28 and acyl ghrelin were assigned using manual integration of the raw data. Amidation (cyan), pyroglutamate (yellow) and octanoylation (purple) post-translational modifications are indicated. GRPP: glicentin-related polypeptide; GLP: glucagon-like peptide; IP: intervening peptide.

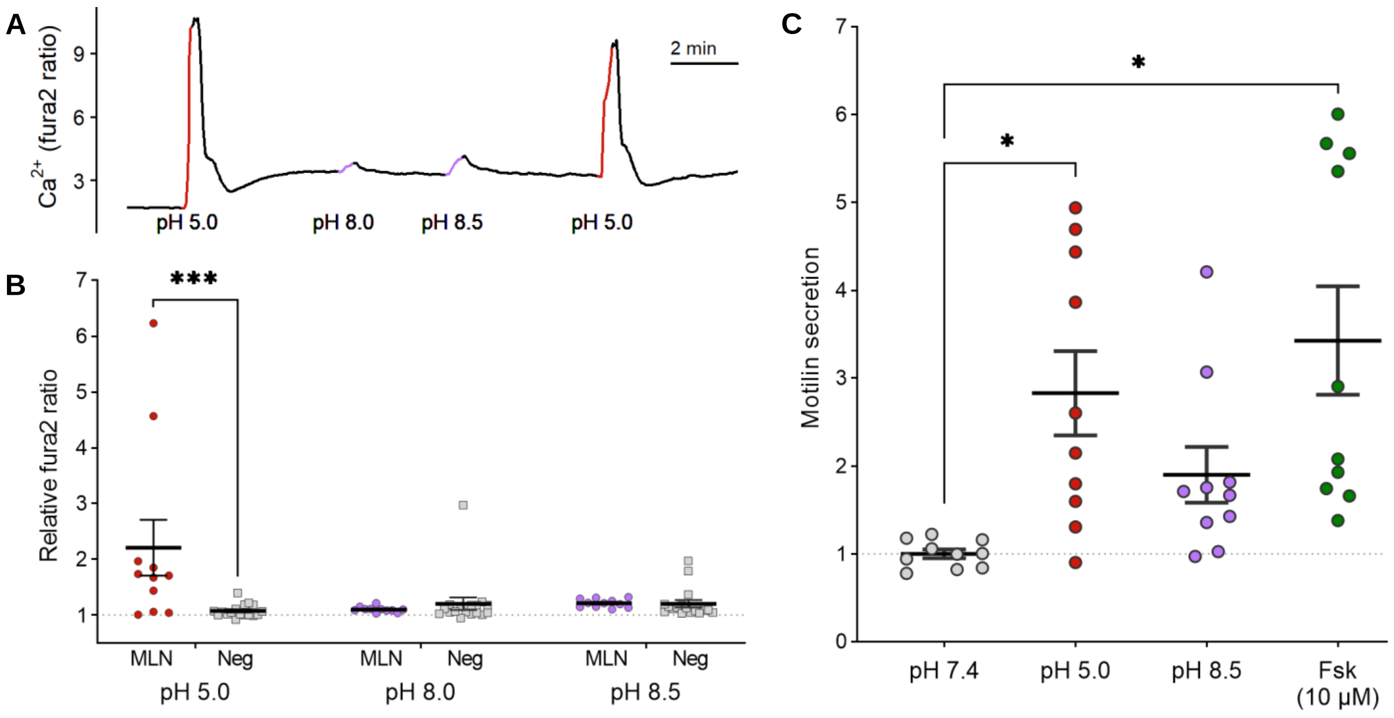

Figure S3 - Effects of alkaline pH

(**A**) Fura2 (340/380 nm) ratio in one representative MLN-Venus cell during perfusion of acidic pH 5.0 (red) or alkaline pH 8.0/8.5 (purple) solutions. (**B**) Data from multiple MLN-Venus and negative (Neg) cells recorded as in (A), as fold change in maximal fura2 ratio. *** $p < 0.001$ by two-way ANOVA ($n = 11$-17 cells from 2 independent experiments). (**C**) Motilin secretion from 2D cultures incubated with acidic (pH 5.0) or alkaline (pH 8.5) saline, expressed relative to basal (pH 7.4) wells measured in parallel, * $p < 0.05$ by Browne- Forsyth and Welch ANOVA with Dunnett's multiple comparisons ($n = 10$ wells from 5 independent experiments).

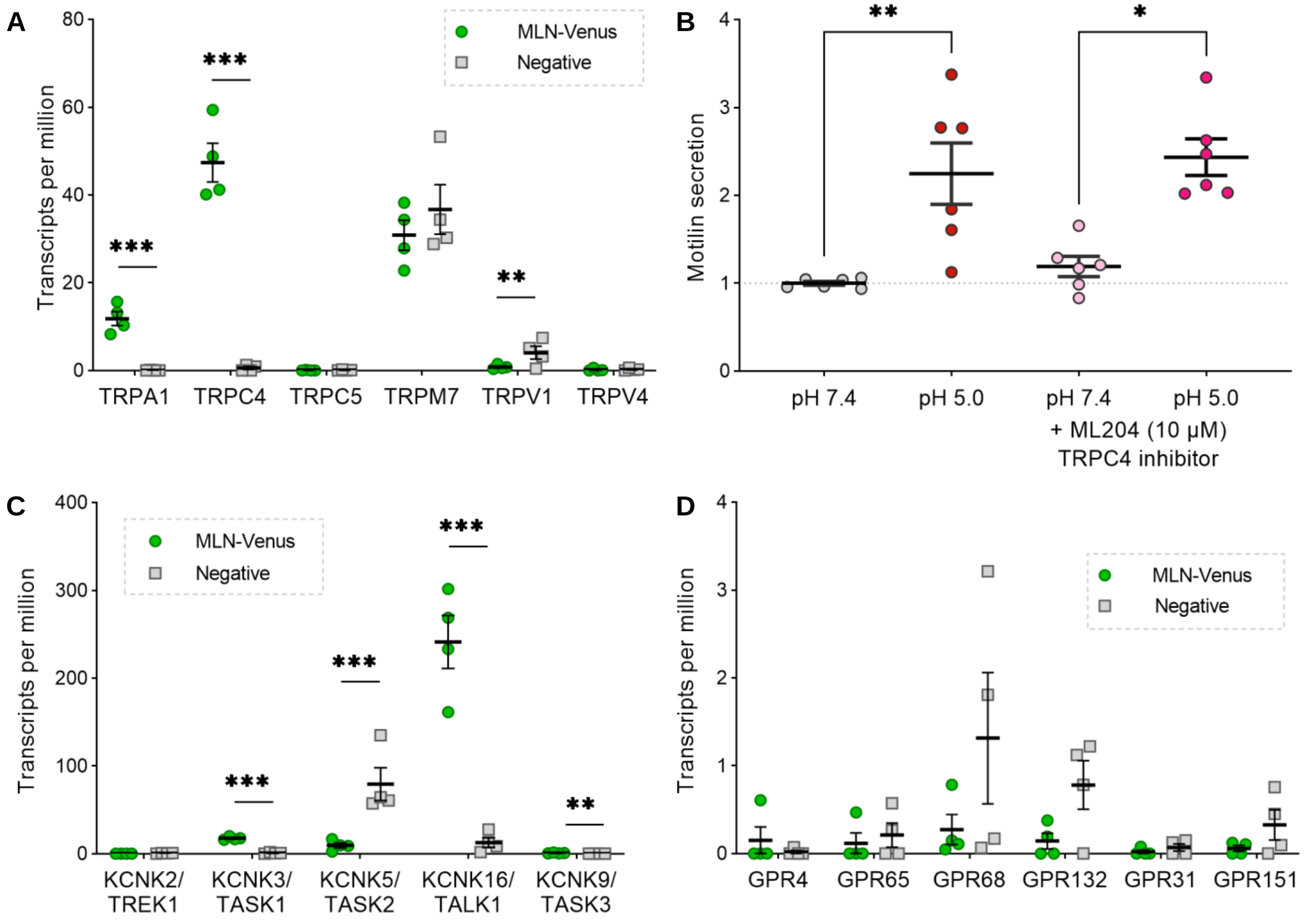

Figure S4 – Expression of acid-responsive machinery in MLN-Venus cells

Bulk RNA sequencing data showing transcripts per million (TPM) of acid-sensitive transient receptor potential channels (**A**), two-pore potassium channels (**C**) and G-protein coupled receptors (**D**) in MLN-Venus and negative cells. (**B**) Motilin secretion in response to pH 5.0 in the presence or absence of TRPC4 inhibitor ML204 (10 µM). * $p < 0.05$, ** $p < 0.01$ by Kruskal-Wallis with Dunn's multiple comparisons test ($n = 6$ wells from 3 independent experiments). Mean ± SEM presented.
